# Bidirectional associations between nightly sleep and daily happiness and negative mood in adolescents

**DOI:** 10.1111/cdev.13798

**Published:** 2022-05-21

**Authors:** Chrystyna D. Kouros, Peggy S. Keller, Olivia Martín‐Piñón, Mona El‐Sheikh

**Affiliations:** ^1^ Department of Psychology Southern Methodist University Dallas Texas USA; ^2^ Department of Psychology University of Kentucky Lexington Kentucky USA; ^3^ Human Development and Family Science Auburn University Auburn Alabama USA

## Abstract

This study examined bidirectional associations between daily happiness and negative mood and subjective and objective sleep measures. Participants were 311 adolescents (*M*_age_ = 17.37 years; 51.8% female; 59.2% White/European American, 38.6% Black/African American, 1% Hispanic/Latinx American, 1.4% multi‐racial; 19.3% below poverty line) observed over a 7‐day period (2017–2018) using sleep diaries and actigraphy. Daily negative mood was related to greater subjective sleep/wake problems, and happiness was related to lower subjective sleep/wake problems. Conversely, shorter self‐reported sleep duration was related to higher negative mood the next day. For actigraphy measures, daily negative mood was related to greater sleep duration and efficiency, whereas happiness was related to lower sleep efficiency. Differences in associations based on subjective versus objective sleep measures are discussed.

Inadequate sleep duration (<8 h) is estimated to affect 56% of U.S. adolescents, with parent‐reported average sleep duration of 7.1 h on school nights (National Sleep Foundation, 2015). Short sleep duration is also related to increases in daytime sleepiness in adolescence compared to childhood (Campbell et al., 2017). Both short sleep duration and poor sleep quality are predictors of internalizing and externalizing symptoms in adolescence (e.g., El‐Sheikh et al., 2020). Although adolescent sleep concerns may be driven by multiple biological and social factors (e.g., puberty‐related shifts in circadian rhythms, time spent engaged with social media or on electronic devices; Hysing et al., 2015; Randler, 2008), there is a growing understanding that mood has an important role to play in adolescent sleep. In particular, adolescent sleep may disrupt or itself be disrupted by adolescent mood. A recent review supports such bidirectional associations between daily sleep and mood generally, but notes that there are few studies of adolescents (Konjarski et al., 2018). The purpose of the current study was to address this gap, examining associations between adolescent daily sleep (objective and subjective measures) and daily happiness and negative mood.

Adolescence is an important developmental period for study because of the normative changes in sleep/wake regulation. In adolescence, there are hormonally driven decreases in the rate of build‐up of the homeostatic drive for sleep, and a delay in the timing of melatonin responsiveness to light dimming in the circadian process of sleep regulation (Hagenauer & Lee, 2013). Although it takes longer to build in adolescence, the homeostatic drive for sleep dissipates at the same rate as in childhood (Crowley et al., 2018). The consequence is that adolescents experience a shift forward in the preferred timing of their sleep rather than a significant decrease in the amount of sleep needed (Fuligni et al., 2019).

Another important reason to focus on nightly sleep and daily mood in adolescence is that brain development during adolescence supports mood regulation and may occur primarily during sleep. During adolescence, there is a 40% decrease in slow wave sleep (Hagenauer & Lee, 2013), which facilitates synaptic pruning and brain reorganization (Feinberg & Campbell, 2010), including in the pre‐frontal cortex (Mallya et al., 2019). Pruning in this region is critical for advancements in emotion regulation (Hare et al., 2008). Brain development in adolescence is also characterized by increased amygdala reactivity to emotional stimuli, and difficulties regulating these intense emotional experiences (Casey & Caudle, 2013). As sleep deprivation increases amygdala reactivity and disrupts connections between the amygdala and regions that regulate emotions, sleep may be especially important for adolescent emotional functioning.

Yet, much of the research on sleep and affective processes is limited to adult humans and animal models. Indeed, a recent review of the research on associations between daily sleep and mood found only six (out of 29 included) studies that examined adolescents (Konjarski et al., 2018), and findings were mixed. For example, in a relatively large sample (*N* = 286) of Dutch adolescents, bidirectional associations between daily self‐reported sleep quality and greater positive and lower negative daily mood were found (van Zundert et al., 2015). In a study of 77 U.S. high school students using objective measures of sleep across 3 nights, Tavernier et al. (2016) found that on days with higher negative self‐evaluative emotions (e.g., embarrassed, ashamed), adolescents experienced longer wake bouts during the night, and on days with higher levels of high‐arousal positive emotions (excited, happy, energetic), adolescents took longer to fall asleep. Given the limited number of actigraphy nights, this study was not able to test for bidirectional associations (i.e., sleep as a predictor of next day affect). Thus, this study provides support for only one direction of effects. In contrast, Doane and Thurston (2014) sampled 78 adolescents across 4 nights using objective measures of sleep and tested both directions of effects; they did not find significant associations between positive affect, negative affect, and any indices of sleep, including sleep duration, sleep efficiency, and time to fall asleep. Models assessing affect, however, also included self‐reported daily stress, which was bidirectionally associated with sleep (i.e., higher daily stress predicted shorter sleep duration that night; reduced sleep efficiency predicted higher daily stress the next day). Since Konjarski et al.’s (2018) review, no additional studies of adolescent daily sleep and mood appear to have been published.

Notably, in a sample of children in middle childhood (approximately 10 years old), Kouros and El‐Sheikh (2015) tested bidirectional associations between child mood and sleep. Children wore an actigraph for seven nights and their parents were called daily to obtain parental reports of child mood. At the within‐person level, only one direction of association was supported: more negative mood during the day was associated with poorer sleep quality (longer sleep latency, greater physical movements) that night. For the opposite direction of association, only between‐person associations were observed. Children who on average exhibited longer sleep latency also reported lower average levels of positive mood during the week. Using self‐reported measures of sleep and mood in a sample of 8‐ to 11‐year‐old children, however, Könen et al. (2016) found support for bidirectional within‐person associations: better sleep quality, but not duration, predicted lower negative affect and higher positive affect the next day, whereas higher evening positive affect predicted better sleep quality that night. Thus, relations in adolescent and younger samples are somewhat less robust compared to findings in adults, for whom associations in both directions of association are typically found (see Konjarski et al., 2018). It is therefore important to evaluate whether patterns of association similar to adults or children are found in adolescence.

For the first direction of effects, we propose that days characterized by greater negative mood (a composite of sadness, anger, worry, frustrated, tension, and irritability) and less happiness than usual will result in nights with lower sleep quality and shorter sleep duration. This hypothesis is based on the understanding that sleep and vigilance are incompatible, and that negative emotional states can promote physiological and cognitive pre‐sleep arousal that interferes with sleep (Harvey, 2002). For example, a comparison of college students classified as good sleepers to those classified as poor sleepers based on self‐report found that poor sleepers reported significantly greater pre‐sleep negative emotions than good sleepers (Ong et al., 2011). Perhaps most compelling, in a study of healthy adults sleeping in a laboratory on a baseline night, a night following a neutral activity, and a night following a failure on a supposed intelligence test, participants experienced greater negative mood on the failure night, as well as polysomnography‐measured reductions in sleep efficiency, sleep duration, and percent of time spent in REM, and increases in the latency to slow wave sleep and the number of awakenings from REM (Vandekerckhove et al., 2011).

The opposite direction of effects is also hypothesized: it is proposed that nights with shorter sleep duration or lower sleep quality than usual will predict days that have greater negative mood and lower happiness. This hypothesis is based on research showing that sleep serves important mood‐regulating functions (Walker & van der Helm, 2009). Neuroimaging studies of healthy adults indicate that sleep deprivation increases the reactivity of the amygdala to emotional stimuli but reduces connectivity between the amygdala and the prefrontal cortex (Yoo et al., 2007), meaning that poor sleep may result in greater emotional reactivity without the ability to regulate those emotions. Indeed, extending the sleep of healthy adult men results in improvement of mood, and these improvements are mediated by prefrontal cortex suppression of amygdala reactivity (Motomura et al., 2017). Although there has been very little research on adolescents, later weekend wake times (suggesting accumulated sleep debt during the school week) are associated with lower gray matter volumes in the amygdala and prefrontal cortex (Lapidaire et al., 2021). Furthermore, following one night of sleep restriction (4 h), healthy adolescents reported more negative mood and anxiety and less positive mood the next day (Reddy et al., 2017).

## Current study

The current study addresses the need for further research on associations between daily mood and sleep among adolescents through inclusion of a large sample, examination of both directions of association, and assessment of sleep using both subjective and objective (actigraphy) measures. Specifically, the purpose of the current study was to test bidirectional associations between daily self‐reported mood, either a negative mood composite (sadness, anger, worry, frustration, tension, and irritability) or happiness, and nightly sleep (subjective sleep duration and quality, actigraphy‐measured sleep duration and efficiency) over 1 week. Both subjective and objective sleep assessment methods have advantages and disadvantages (Sadeh, 2015), and multi‐method assessments are key for explicating relations between sleep and developmental outcomes. Whereas self‐reports of sleep provide unique and important information about one's subjective sleep experience (e.g., feeling tired) and whether objective sleep parameters satisfy individual sleep needs, it is also important to include objective measures to reduce confounds related to retrospective reporting and/or mono‐method reporting bias. Critically for studies also assessing affect, objective measures of sleep can reduce mood‐congruent bias (i.e., assuming one slept well or poorly based on their mood) (Doane & Thurston, 2014; Konjarski et al., 2018). We considered both sleep duration and quality. Specifically, we included self‐reported and actigraphy‐based sleep minutes (sleep duration), and self‐reported sleep/wake problems and actigraphy‐based sleep efficiency (sleep quality). In confirmatory analyses (i.e., analyses with directional hypotheses), we expected that lower than usual happiness and higher than usual negative mood would be associated with more sleep problems that night (shorter duration, worse sleep quality). We also hypothesized that shorter than usual sleep duration and worse sleep quality than usual would predict lower levels of daily happiness and higher levels of negative mood the next day.

To provide more cogent conclusions about associations between sleep and daily mood, we considered several daily‐level covariates that may be associated with either sleep or daily mood, including being sick, in pain, and whether participants slept in their own beds (e.g., Tamaki et al., 2016). Also increasing the robustness of tests, and consistent with prior sleep studies with adolescents, analyses controlled for between‐person differences in child sex, race and ethnicity, and socioeconomic status (SES; operationalized as income‐to‐needs ratio) that have been associated with adolescent mood and sleep problems (e.g., El‐Sheikh et al., 2020; Guglielmo et al., 2018; Shimizu et al., 2020; Yip et al., 2020).

Whereas biological process underlie both sleep and mood, social and cultural factors are also important processes that may moderate the bidirectional associations between sleep and mood. Therefore, in post hoc, exploratory analyses, we also tested sex, race and ethnicity, and SES as potential moderators of within‐person associations between sleep and mood. Notably, Biggs et al. (2013) tested sex, race/ethnicity, and SES differences in parent‐reported child sleep in a sample of Australian children and found that non‐White children (compared to White children) and children from lower SES (compared to higher SES) reported less sleep. A limitation of this study, however, was the uneven sample sizes between the White (85% of sample) and non‐White groups. Furthermore, the authors found that girls spent more time in bed than boys. A narrative review of 23 studies on racial/ethnic disparities in sleep in adolescent samples found that the majority of studies reported that White youth had longer sleep duration compared to Black and Hispanic youth, but evidence for health disparities in sleep/wake problems was mixed (Guglielmo et al., 2018). Whereas sleep disparities based on demographic factors have been examined, it remains unclear whether associations between sleep and mood also differ. Indeed, Könen et al.'s (2016) systematic review of prospective studies on sleep and daily mood associations noted that associations may differ based on demographic characteristics such as sex and race; yet empirical tests of individual differences based on demographic characteristics remain a gap in this area of research. Grandner et al.'s (2010) socioecological model of sleep and health highlights that there are multiple levels of influence including both proximal and distal processes. Cultural and socioeconomic factors can affect beliefs and attitudes about sleep as well as the physical sleep environment, thereby serving as individual difference variables that may directly predict and also moderate associations between sleep and psychological outcomes (Grandner et al., 2010). Given the size and diversity of our sample, our study was uniquely positioned to address this gap in the literature.

## METHOD

### Participants

Participants were 323 youth and their families from the fourth wave (W4) of a longitudinal study that focuses on sleep and health (Auburn University Sleep Study). Data for W4 were collected during 2017 and 2018. We selected data from W4 for the current study given our focus on the developmental period of adolescence; of note, there was a 6‐year time lag between W3 and W4. Most adolescents (61.6%) participated in previous study waves (W1‐W3, 2009–2012) and 124 participated for the first time in the current wave. Recruitment strategies and locale were the same for all youth. Letters were distributed at public schools in small towns in the southeastern United States and interested families contacted our laboratory. Youth with a diagnosed sleep disorder or learning disability based on mothers' reports were not eligible. More information about recruitment and the initial study waves is provided in Shimizu et al., 2020. The analytic sample was composed of 311 adolescents who completed the actigraphy protocol for this study (described below). On average, adolescents were 17.37 years old (*SD* = 0.87 years) and 51.8% were female. Youth were diverse in relation to race and ethnicity (59.2% White/European American, 38.6% Black/African American, 1% Hispanic/Latinx American, and 1.4% multi‐racial). The average income‐to‐needs ratio, calculated from parent‐report of family income and number of people living in the household, was 2.79 (*SD* = 1.97). An income‐to‐needs ratio of <1 was considered below the poverty line (19.3% of participants), 1–2 was considered at or near the poverty line (19.3%), 2–3 was considered lower middle class (20.1%), 3–4 was considered middle class (19.3%), and ≥4 was considered upper middle class (22.0% of participants).

### Procedures

All procedures were approved by the university's institutional review board, and parent consent and adolescent assent were obtained. Demographic information about participants (e.g., age, ethnicity) was collected from mothers during a pre‐study phone interview. Sleep data were collected during the regular school year, excluding holidays. Actigraphs were mailed to the adolescents' homes and they were instructed to wear them on their non‐dominant wrist for seven consecutive nights. Youth completed sleep logs and sleep diaries for each night of actigraphy to corroborate objective sleep data with self‐reported bed and wake times as well as to report daily mood. All measures were completed in English.

### Measures

#### Adolescent negative mood and happiness

Each night that adolescents wore the actigraph, they also completed a daily sleep survey, in which they rated how they felt that day on a 5‐point Likert scale (1 = *not at all*, 5 = *extremely*). A negative mood composite was created by summing responses on the following items: sad, angry, worried, frustrated, tense, and irritable. Following recommendations by Cranford et al. (2006), both the between‐ (R_1F_ = 0.86) and within‐ (R_C_ = 0.73) person reliability of this daily negative mood composite were acceptable. Reliabilities calculated separately for White/European American (R_1F_ = 0.88, R_C_ = 0.74) and Black/African American (R_1F_ = 0.80, R_C_ = 0.72) groups were also acceptable. Daily happiness was assessed with adolescents' ratings on one item in which they rated how happy they felt today on the same 5‐point Likert scale.

#### Subjective measures of sleep

Adolescents reported their bedtime (“What time did you go to sleep last night?”) and wake time (“What time did you wake up this morning?”) on their daily sleep diary. The time between their bedtime and wake time the next day was calculated for *self‐reported sleep minutes*. A *subjective sleep/wake problems composite* was created by summing adolescents' daily reports on the following five questions from the sleep diary: 1) When you woke up was it (answer choices: very easy, easy, neither easy nor hard, very hard); 2) When you first woke up did you feel (answer choices: wide awake, fairly awake, neutral, somewhat sleepy, very sleepy); 3) After waking, did you feel (answer choices: very rested, somewhat rested, okay, somewhat not rested, not rested at all); 4) Overall, was the quality of your sleep (answer choices: excellent, good, average, fair, or poor); and 5) In general, today did you (answer choices: feel alert, feel somewhat sleepy, doze off, fall asleep). Higher scores reflect higher levels of sleep/wake problems (i.e., poorer sleep quality). The between‐ (R_1F_ = 0.76) and within‐ (R_C_ = 0.64) person reliability of the daily subjective sleep/wake problems composite were acceptable. Reliabilities calculated separately for White/European American (R_1F_ = 0.76, R_C_ = 0.61) and Black/African American (R_1F_ = 0.75, R_C_ = 0.67) groups were also acceptable.

#### Objective measures of sleep

To mirror our self‐report assessment, and reduce type I error, we selected one well‐established actigraphy‐based indicator each of sleep quantity and sleep quality. Actigraphy data were used to derive objective measures of sleep minutes and sleep efficiency, through Octagonal Basic Motionloggers (Ambulatory Monitoring Inc., Ardsley, NY). The motion was measured in 1‐min epochs using a zero‐crossing mode. Raw data were analyzed with the ACTme software (Action W2, 2002, Ambulatory Monitoring, Inc., Ardsley, NY) and using Sadeh's scoring algorithm (Sadeh et al., 1994). Consistent with the manual that accompanied the software, *sleep minutes* were operationalized as the amount of lapsed time between sleep onset and morning wake time excluding awakenings, and *sleep efficiency* was calculated as the percentage of epochs scored as sleep between sleep onset and wake time. Sleep onset was determined by the first minute of the first three consecutive minutes scored as sleep and sleep offset was determined by the last minute of the last five consecutive minutes scored as sleep by the ACTme software program. Per best practices, one experienced coder manually set the beginning and end of sleep intervals based on the amount of movement detected by the actigraph's accelerometer. To corroborate these times, we used participants' sleep logs in which they self‐reported their bed and wake times. This method is consistent with coding instructions used in several prior studies (e.g., El‐Sheikh et al., 2022), the Acebo and Carskadon's (2001) laboratory manual, and with standard setup for the interval criteria (Sadeh et al., 1994). Once the interval was set, the software algorithm generated all values for *sleep minutes* and *sleep efficiency*. Nights of data were excluded when sleep onset and wake times reported in the sleep logs differed from actigraphy‐determined times by more than 30 minutes, which happened infrequently because participants were instructed to wear the actigraph as they were getting into bed to sleep and to remove it immediately after waking. Participants also pressed an event marker on the actigraph to indicate first attempt to fall asleep. Thus, the sleep onset times from the sleep logs and determined by the actigraphy were very similar, as evidenced by only 5.28 adolescents, on average, being excluded per night for more than a 30‐minute discrepancy. Together, sleep minutes and sleep efficiency are two of the most commonly studied sleep indices in investigations of reciprocal associations between sleep and daily mood and capture both sleep duration and quality (Konjarski et al., 2018).

#### Potential daily covariates

Adolescents also reported on daily experiences that may be associated with sleep or their daily mood on their daily sleep diary. Specifically, adolescents responded (yes/no) to the following: “Were you sick yesterday?”, “Were you experiencing any pain yesterday?”, and “Did you sleep in your own bed last night?”. Each response was coded 0 = no and 1 = yes.

### Analysis plan

We used multilevel modeling to test for within‐person associations between daily happiness and negative mood and both subjective and objective measures of sleep duration and quality. Specifically, we tested the extent to which within‐person fluctuations in daily happiness and negative mood predicted adolescents' sleep that night, as well as the extent to which within‐person fluctuations in sleep duration and quality predicted adolescents' happiness and negative mood the next day. There were a total of 8 models that tested mood as a predictor of sleep (i.e., 4 models in which happiness predicted each sleep outcome and 4 models in which negative mood predicted each sleep outcome). There were also eight models that tested sleep as a predictor of happiness or negative mood on the next day (4 models in which each sleep variable predicted happiness and 4 models in which each sleep variable predicted negative mood). Following centering guidelines for disentangling within‐person and between‐person effects (Bolger & Laurenceau, 2013), we person‐centered all Level 1 predictors of sleep or mood, such that they represented within‐person fluctuations from one's usual level. We also included each person's average score across the actigraphy/diary period as a predictor of the intercept in Level 2 models to account for between‐person associations between mood and sleep. When mood was the dependent variable (i.e., sleep predicting the next day's mood), we controlled for the autoregressive effect of mood on the previous day at Level 1. An a priori decision was made to allow for random slopes at Level 2 given we had a sufficient number of diary days (i.e., degrees of freedom) to model these effects and, theoretically, the assumption of individual differences in sleep‐mood associations is more plausible than the assuming there are no individual differences. In preliminary analyses, we tested the extent to which mood and sleep systematically varied depending on the day of the reporting period (first day of actigraphy/diary collection coded 0) and whether it was a weekday (i.e., school night) or weekend night (Sunday through Thursday coded 0, Friday and Saturday coded 1); these were included as predictors at Level 1.

We also considered the following potential daily confounds on adolescents' mood and sleep at Level 1: adolescents' daily reports of being sick, experiencing pain, or sleeping in their own bed that day. We also accounted for between‐person differences in baseline (i.e., day 1) sleep and mood based on sex, race and ethnicity, and SES by including these as predictors of the intercept in models at Level 2. Sex was coded such that 0 = female and 1 = male, and race/ethnicity was coded 0 = White/European American and 1 = racial or ethnic minority (Black/African America, Hispanic/Latinx American, or multi‐racial). SES was operationalized as income‐to‐needs ratio and was entered in models grand‐mean centered. Multilevel modeling analyses were conducted using Hierarchical Linear Modeling (HLM) v. 8.1 software (Raudenbush et al., 2019). Models that included objective sleep measures from actigraphy data as the dependent variable were based on all 311 adolescents; 2 adolescents, however, did not have complete sleep diaries and therefore analyses in which subjective sleep or mood was the dependent variable are based on a sample size of 309. Supplemental, post hoc analyses examined moderation of associations by adolescent sex, race and ethnicity, and SES.

## RESULTS

Based on the sleep and mood variables averaged across the 7 days, and using Tabachnick and Fidell's (2012) recommendation of 3.29 *SD* from the mean, very few outliers were identified (range: 0% to 2.3%). All potential outliers were within range and plausible values; therefore, no data were excluded from analyses. Q‐Q plots indicated that the sleep and mood variables were normally distributed, with the exceptions of objective sleep efficiency, which showed slight negative skew (skewness = −1.84), and self‐reported negative mood, which slowed slight positive skew (skewness = 1.16). Given skewness estimates were within ±2 (George & Mallory, 2010), the normality assumption was reasonable for these data. Furthermore, simulation studies show that multilevel models are robust to violations of normality, producing unbiased parameter estimates for fixed effects (Maas & Hox, 2004).

On average, adolescents had 5.96 nights of actigraphy data (*SD* = 1.20); 86.5% of adolescents had at least 5 nights worth of actigraphy data and 44.7% of adolescents had valid actigraphy data for all 7 nights. Of the 309 adolescents who completed the sleep log and diaries, 97.4% completed all 7 nights. Notably, multilevel modeling can accommodate missing data at Level 1; therefore, all available data from participants were used in analyses. Table 1 provides descriptive information for the objective and subjective sleep measures and self‐reported mood, averaged across the 7 days. Adolescents self‐reported an average bed time of 11:00 pm and a wake time of 7:07 am. Actigraphy‐determined bed time, on average, was 11:37 pm and wake time was 6:53 am. Based on self‐reports, adolescents' average nightly sleep duration across the week was 8.12 h (*SD* = 1.28 h), and based on actigraphy data, the average nightly sleep duration was 6.74 h (*SD* = 1.01 h). The difference between self‐reported and actigraphy‐measured average nightly sleep duration was significant, *t*(298) = 19.54, *p* < .001. Bivariate associations between self‐reported and objective sleep minutes ranged from *r* = .36–.57 (all *p* < .001) across the week. With regard to sleep quality, self‐reported sleep/wake problems were not significantly associated with objective sleep efficiency on any of the diary days.

**TABLE 1:** Descriptive information on study variables averaged across days

	ICC	*M*	*SD*	Range
Self‐reported negative mood composite	0.60	1.75	0.62	1–4.25
Self‐reported happiness	0.46	3.74	0.68	1.57–5
Self‐reported sleep minutes	0.27	487.43	76.89	179–790
Self‐reported subjective sleep/wake problems	0.46	12.03	2.97	4.17–22.83
Objective sleep minutes	0.35	404.46	60.77	214–614.43
Objective sleep efficiency	0.57	92.87	6.73	62.91–100

### Preliminary findings

Initial multilevel models were conducted for each mood and sleep variable, with Time and Weekend as Level 1 predictors to test the extent to which mood or sleep systematically changed during the course of the week or differed on weekends as compared to during the week (Table 2, Model 1). Both mood variables and all sleep variables were significantly different on the weekends (Friday, Saturday) as compared to the weekdays. Specifically, levels of negative mood were lower, b = −0.07, *SE* = 0.02, *p* = .005, and happiness was higher, b = 0.10, *SE* = 0.03, *p* = .002, on the weekend. Additionally, weekend nights were associated with longer sleep duration (self‐reported sleep minutes: b = 41.50, *SE* = 7.12, *p* < .001; objective sleep minutes, b = 26.66, *SE* = 4.44, *p* < .001) and fewer subjective sleep/wake problems, b = −0.69, *SE* = 0.16, *p* < .001. Objective sleep efficiency, however, was higher during the week as compared to the weekend, b = −0.63, *SE* = 0.32, *p* = .049. Based on recommendations for working with intensive longitudinal data (Bolger & Laurenceau, 2013), time and weekend were included in subsequent models to account for any unmeasured third‐variable confounds that are associated with time or day of the week.

**TABLE 2:** Preliminary models testing time, weekend, and potential confounding variables of sleep and mood

	Mood		Sleep parameter			
	Negative mood	Happiness	Self‐reported sleep minutes	Self‐reported sleep/wake problems	Objective sleep minutes	Objective sleep efficiency
	b (SE), *p*	b (SE), *p*	b (SE), *p*	b (SE), *p*	b (SE), *p*	b (SE), *p*
Model 1						
Intercept	1.80 (0.04), *p* < .001	3.69 (0.04), *p* < .001	487.83 (6.07), *p* < .001	12.27 (0.20), *p* < .001	403.53 (4.48), *p* < .001	92.97 (0.43), *p* < .001
Time	−0.01 (0.005), *p* = .06	0.01 (0.01), *p* = .32	−4.50 (1.53), *p* = .004	−0.07 (0.03), *p* = .054	−1.78 (0.86), *p* = .038	0.03 (0.07), *p* = .66
Weekend	−0.07 (0.02), *p* = .005	0.10 (0.03), *p* = .002	41.50 (7.12), *p* < .001	−0.69 (0.16), *p* < .001	26.66 (4.44), *p* < .001	−0.63 (0.32), *p* = .049
Model 2						
Intercept	1.76 (0.06), *p* < .001	3.72 (0.07), *p* < .001	481.53 (12.61), *p* < .001	12.21 (0.33), *p* < .001	394.83 (8.83), *p* < .001	93.45 (0.75), *p* < .001
Time	−0.004 (0.01), *p* = .46	0.01 (0.01), *p* = .30	−4.48 (1.53), *p* = .004	−0.02 (0.04), *p* = .50	−1.34 (0.92), *p* = .15	−0.03 (0.07), *p* = .67
Weekend	−0.06 (0.03), *p* = .013	0.09 (0.03), *p* = .012	45.75 (7.41), *p* < .001	−0.72 (0.17), *p* < .001	29.60 (4.68), *p* < .001	−0.48 (0.34), *p* = .16
Sick	−0.01 (0.06), *p* = .82	−0.14 (0.09), *p* = .11	1.93 (11.04), *p* = .86	0.06 (0.32), *p* = .86	11.43 (11.84), *p* = .34	−1.43 (0.76), *p* = .06
Pain	0.16 (0.05), *p* < .001	−0.12 (0.06), *p* = .048	−3.12 (8.05), *p* = .70	0.87 (0.27), *p* < .001	−0.45 (6.32), *p* = .94	−1.39 (0.52), *p* = .008
Slept in Own Bed	−0.01 (0.05), *p* = .88	−0.01 (0.06), *p* = .85	6.60 (11.69), *p* = .57	−0.13 (0.29), *p* = .65	7.62 (8.23), *p* = .36	−0.20 (0.67), *p* = .77

*Note*: Estimates with robust standard errors reported. Time coded so that actigraphy/diary day 1 = 0. Weekend coded as 1 = weekend and 0 = weekday. Sick, Pain, and Slept in own bed coded as 0 = no and 1 = yes.

Next, toward stringent assessment of the research questions, self‐reports on sleep diaries of whether the adolescent was sick, experiencing pain, and slept in their own bed that night (all coded 0 = no and 1 = yes) were added to models to test whether they were associated with daily mood or sleep and should be included as daily‐level covariates in subsequent models (Table 2, Model 2). Neither feeling sick nor sleeping in their own bed was significantly associated with adolescents' mood or any of the sleep variables. Experiencing pain, however, was related to higher levels of negative mood, b = 0.16, *SE* = 0.05, *p* < .001, and lower levels of happiness, b = −0.12, *SE* = 0.06, *p* = .048. Experiencing pain was also associated with high levels of subjective sleep/wake problems, b = 0.87, *SE* = 0.27, *p* < .001, and less objective sleep efficiency, b = −1.39, *SE* = 0.52, *p* = .008. Pain was not associated with self‐reported or objective sleep minutes. Based on these initial findings, daily pain was included as a within‐person covariate in all models, along with between‐person covariates identified as important in prior research (adolescent sex, race and ethnicity, and SES).

### Associations between daily negative mood and sleep that night

Results from models testing within‐person associations between fluctuations in daily negative mood and sleep the same night are presented in Table 3. There were significant within‐person associations between negative mood and all sleep indices, with the exception of self‐reported sleep minutes. Specifically, days with greater negative mood than usual were associated with higher levels of self‐reported sleep/wake problems, b = 1.30, *SE* = 0.15, *p* < .001, but also greater objective sleep efficiency, b = 0.72, *SE* = 0.30, *p* = .015, and more objective sleep minutes, b = 7.20, *SE* = 3.62, *p* = .048, that night. There was also a significant between‐person association between negative mood and self‐reported sleep/wake problems, b = 2.17, *SE* = 0.28, *p* < .001, indicating that individuals who, on average, had higher levels of negative mood also reported, on average, higher levels of sleep/wake problems.

**TABLE 3:** Within‐person fluctuations in daily negative mood as predictor of subjective and objective measures of sleep the same night

	Dependent variable			
	Self‐reported sleep minutes	Self‐reported sleep/wake problems	Objective sleep minutes	Objective sleep efficiency
	Fixed effects			
	b (SE), *p*	b (SE), *p*	b (SE), *p*	b (SE), *p*
Intercept	487.41 (6.50), *p* < .001	12.47 (0.18), *p* < .001	402.30 (486), *p* < .001	93.13 (0.44), *p* < .001
Time	−4.17 (1.62), *p* = .011	−0.10 (0.03), *p* = .002	−1.12 (0.94), *p* = .24	−0.01 (0.07), *p* = .89
Weekend vs. weekday	41.95 (7.42), *p* < .001	−0.57 (0.15), *p* < .001	27.76 (4.71), *p* < .001	−0.36 (0.33), *p* = .29
Daily pain	−0.72 (8.77), *p* = .94	0.55 (0.22), *p* = .012	−1.57 (6.50), *p* = .81	−1.71 (0.54), *p* = .002
Negative mood_PC_ (within‐person)	0.67 (4.91), *p* = .89	**1.30 (0.15), *p* < .001**	**7.20 (3.62), *p* = .048**	**0.72 (0.30, *p* = .015**
Average negative mood (between‐person)	−7.87 (6.59), *p* = .23	**2.17 (0.28), *p* < .001**	−3.03 (5.25), *p* = .56	0.05 (0.64), *p* = .94
Sex	−14.00 (8.62), *p* = .11	−0.17 (0.28), *p* = .55	−24.09 (6.65), *p* < .001	−1.68 (0.76), *p* = .028
Race/ethnicity	−24.44 (8.84), *p* = .006	−0.42 (0.32), *p* = .19	−32.12 (7.65), *p* < .001	−2.44 (0.87), *p* ‐ .005
Income‐to‐needs ratio	−3.74 (2.34), *p* = .11	0.08 (0.10), *p* = .44	2.66 (1.77), *p* = .13	0.63 (0.19), *p* = .001
	Random effects (Variance estimates)			
Level 2 (Between‐person)				
Intercept	5786.12	5.70***	3355.52**	36.11
Time	130.05	0.02**	19.17**	0.13
Weekend	6724.45*	2.10**	1781.89***	4.58
Pain	3575.63	1.05*	740.08*	5.18
Negative mood	173.79	1.26	77.58	2.22

* *p* < .05

** *p* < .01

*** *p* < .001.

*Note*: Results from models with robust standard errors reported. Bold indicates a significant within‐person or between‐person association between negative mood and sleep. PC, person‐centered. Weekend coded 0 = weekday, 1 = weekend; Pain coded 0 = no, 1 = yes; Sex coded 0 = female, 1 = male; Race/ethnicity coded 0 = White/European American, 1 = racial/ethnic minority.

### Associations between daily happiness and sleep that night

Results from models testing within‐person associations between fluctuations in daily happiness and sleep the same night are presented in Table 4. Significant within‐ and between‐person associations emerged between daily happiness and self‐reported sleep/wake problems. Days with higher than usual happiness were associated with fewer self‐reported sleep/wake problems that night, b = −0.87, *SE* = 0.10, *p* < .001. At the between‐person level, adolescents who, on average, had higher levels of happiness, also reported, on average, fewer sleep/wake problems, b = −1.71, *SE* = 0.25, *p* < .001. There was also a significant within‐person association between daily happiness and objective sleep efficiency. Contrary to hypotheses, higher than usual happiness was associated with less sleep efficiency that night, b = −0.48, *SE* = 0.24, *p* = .045. There were no significant within‐ or between‐person associations between daily happiness and subjective or objective sleep minutes the same night.

**TABLE 4:** Within‐person fluctuations in daily happiness as predictor of subjective and objective measures of sleep the same night

	Dependent variable			
	Self‐reported sleep minutes	Self‐reported sleep/wake problems	Objective sleep minutes	Objective sleep efficiency
	Fixed effects			
	b (SE), *p*	b (SE), *p*	b (SE), *p*	b (SE), *p*
Intercept	487.30 (6.50), *p* < .001	12.44 (0.18), *p* < .001	402.11 (4.87), *p* < .001	93.12 (0.44), *p* < .001
Time	−4.09 (1.63), *p* = .012	−0.09 (0.03), *p* = .003	−1.11 (0.95), *p* = .24	−0.01 (0.07), *p* = .87
Weekend vs. weekday	42.47 (7.41), *p* < .001	−0.58 (0.15), *p* < .001	28.11 (4.73), *p* < .001	−0.40 (0.33), *p* = .23
Daily pain	−0.23 (8.58), *p* = .98	0.87 (0.23), *p* < .001	−0.55 (6.50, *p* = .93	−1.53 (0.54), *p* = .005
Daily happiness_PC_ (within‐person)	2.57 (3.79), *p* = .50	**−0.87 (0.10), *p* < .001**	−4.56 (2.87), *p* = .11	**−0.48 (0.24), *p* = .045**
Averaged happiness (between‐person)	1.33 (7.74), *p* = .86	**−1.71 (0.25), *p* < .001**	−2.52 (5.44), *p* = .64	0.04 (0.53), *p* = .94
Sex	−12.60 (8.39), *p* = .13	−0.49 (0.29), *p* = .088	−22.54 (6.58), *p* = .001	−1.60 (0.75), *p* = .033
Race/ethnicity	21.97 (8.79), *p* = .013	−0.81 (0.33), *p* = .014	−30.66 (7.46), *p* < .001	−2.29 (0.85), *p* = .008
Income‐to‐needs ratio	−3.37 (2.37), *p* = .16	0.04 (0.10), *p* = .66	3.03 (1.84), *p* = .10	0.60 (0.19), *p* = .002
	Random effects (Variance estimates)			
Level 2 (Between‐person)				
Intercept	5815.07	3.20***	3356.17*	35.70**
Time	138.59	0.02*	17.67**	0.07
Weekend	6579.88***	2.39***	1812.27***	3.92
Pain	3066.23*	1.32	741.36	6.72
Positive mood	99.06	0.44*	58.99	1.75

* *p* < .05

** *p* < .01

*** *p* < .001.

*Note*: Results from models with robust standard errors reported. Bold indicates a significant within‐person or between‐person association between negative mood and sleep. PC, person centered. Weekend coded 0 = weekday, 1 = weekend; Pain coded 0 = no, 1 = yes; Sex coded 0 = female, 1 = male; Race/ethnicity coded 0 = White/European American, 1 = racial/ethnic minority.

### Associations between sleep and negative mood the next day

Results from models testing within‐person associations between fluctuations in sleep duration or quality and negative mood the next day are presented in Table 5. As before, analyses controlled for day of the actigraphy data collection period, whether it was a weekend or weekday, and self‐reported pain that day at Level 1. Analyses also controlled for the autoregressive effect of the previous days' mood at Level 1. At Level 2, models accounted for child sex, race and ethnicity, and the family's income‐to‐needs ratio. There was one significant within‐person association between fewer than usual self‐reported sleep minutes and an increase in negative mood from one day to the next, b = −0.0003, *SE* = 0.0002, *p* = .049. A between‐person association emerged, showing that higher average levels of self‐reported sleep/wake problems were associated with higher average levels of negative mood, b = 0.07, *SE* = 0.01, *p* < .001. No significant within‐ or between‐person associations were found between objective indicators of sleep duration or quality and negative mood on the next day.

**TABLE 5:** Within‐person fluctuations in sleep as predictor of negative mood the next day

	Dependent variable: negative mood			
	Self‐reported sleep minutes predicting negative mood	Self‐reported sleep/wake problems predicting negative mood	Objective sleep minutes Predicting negative mood	Objective sleep efficiency predicting negative mood
	Fixed effects			
	b (SE), *p*	b (SE), *p*	b (SE), *p*	b (SE), *p*
Intercept	1.90 (0.04), *p* < .001	1.88 (0.03), *p* < .001	1.90 (0.09), *p* < .001	1.91 (0.04), *p* < .001
Time	−0.05 (0.01), *p* < .001	−0.05 (0.01), *p* < .001	−0.05 (0.01), *p* < .001	−0.05 (0.01), *p* < .001
Weekend vs. Weekday	−0.07 (0.03), *p* = .024	−0.08 (0.03), *p* = .007	−0.09 (0.03), *p* = .009	−0.09 (0.03), *p* = .005
Daily pain	0.09 (0.05), *p* = .075	0.09 (0.05), *p* = .083	0.08 (0.05), *p* = .14	0.08 (0.05), *p* = .14
Previous day negative mood	0.20 (0.03), *p* < .001	0.15 (0.03), *p* < .001	0.24 (0.03), *p* < .001	0.24 (0.03), *p* < .001
Sleep_PC_ (within‐person)	**−0.0003 (0.0002), *p* = .049**	0.001 (0.01), *p* = .79	−0.0003 (0.0002), *p* = .19	−0.001 (0.002), *p* = .71
Averaged Sleep (between‐person)	−0.0002 (0.0003), *p* = .60	**0.07 (0.01), *p* < .001**	−0.0001 (0.0004), *p* = .86	0.002 (0.004), *p* = .56
Sex	−0.20 (0.05), *p* < .001	−0.17 (0.05), *p* < .001	−0.22 (0.05), *p* < .001	−0.22 (0.05), *p* < .001
Race/ethnicity	−0.22 (0.06), *p* < .001	−0.14 (0.05), *p* = .013	−0.19 (0.06), *p* = .02	−0.19 (0.06), *p* = .002
Income‐to‐needs ratio	−0.03 (0.01), *p* = .018	−0.03 (0.01), *p* = .044	−0.03 (0.01), *p* = .014	−0.04 (0.01), *p* = .013
	Random effects (Variance estimates)			
Level 2				
Intercept	0.18	0.14	0.17**	0.17**
Time	0.003	0.002	0.003**	0.003
Weekend	0.02	0.03*	0.04*	0.03*
Pain	0.08	0.09	0.06	0.06
Previous day negative mood	0.08	0.07	0.08***	0.09**
Sleep	0.000**	0.001*	0.000**	0.0001*

* *p* < .05

** *p* < .01

*** *p* < .001; The significant level 2 random effects that are reported as 0.000 are not in fact zero, but are so small that they round to this value.

*Note*: Results from models with robust standard errors reported. Bold indicates a significant within‐person or between‐person association between negative mood and sleep. PC = person‐centered. Weekend coded 0 = weekday, 1 = weekend; Pain coded 0 = no, 1 = yes; Sex coded 0 = female, 1 = male; Race/ethnicity coded 0 = White/European American, 1 = racial/ethnic minority.

### Associations between sleep and happiness the next day

Results from models testing within‐person associations between fluctuations in sleep duration or quality and happiness the next day are presented in Table 6. There were no significant within‐person associations between self‐reported or objective indicators of sleep and levels of happiness on the next day. There was, however, one significant between‐person association, such that adolescents who, on average, reported higher levels of sleep/wake problems also reported lower average levels of happiness, b = −0.07, *SE* = 0.01, *p* < .001.

**TABLE 6:** Within‐person fluctuations in sleep as predictor of happiness the next day

	Dependent variable: Daily happiness			
	Self‐reported sleep minutes predicting happiness	Self‐reported sleep/wake problems predicting happiness	Objective sleep minutes predicting happiness	Objective sleep efficiency predicting happiness
	Fixed effects			
	b (SE), *p*	b (SE), *p*	b (SE), *p*	b (SE), *p*
Intercept	3.71 (0.04), *p* < .001	3.72 (0.03), *p* < .001	3.52 (0.12), *p* < .001	3.71 (0.05), *p* < .001
Time	0.002 (0.01), *p* = .83	0.01 (0.01), *p* = .61	−0.002 (0.01), *p* = .90	−0.002 (0.01), *p* = .87
Weekend vs weekday	0.10 (0.04), *p* = .018	0.10 (0.04), *p* = .019	0.12 (0.04), *p* = .008	0.11 (0.04), *p* = .011
Daily pain	−0.13 (0.06), *p* = .033	−0.10 (0.06), *p* = .095	−0.12 (0.06), *p* = .065	−0.13 (0.06), *p* = .049
Previous day happiness	0.11 (0.03), *p* < .001	0.06 (0.03), *p* = .057	0.13 (0.03), *p* < .001	0.13 (0.03), *p* < .001
Sleep_PC_ (within‐person)	−0.00003 (0.00002), *p* = .88	0.004 (0.008), *p* = .60	0.0001 (0.0003), *p* = .83	0.0003 (0.004), *p* = .94
Averaged sleep (between‐person)	−0.0001 (0.001), *p* = .80	**−0.07 (0.01), *p* < .001**	−0.001 (0.001), *p* = .084	−0.007 (0.01), *p* = .23
Sex	0.08 (0.07), *p* = .23	0.06 (0.06), *p* = .39	0.06 (0.07), *p* = .40	0.08 (0.07), *p* = .26
Race/ethnicity	0.06 (0.07), *p* = .45	0.0004 (0.07), *p* = .99	0.02 (0.07), *p* = .83	0.04 (0.08), *p* = .62
Income‐to‐needs ratio	0.05 (0.02), *p* = .003	0.05 (0.02), *p* = .008	0.05 (0.02), *p* = .003	0.05 (0.02), *p* = .002
	Random effects (Variance estimates)			
Level 2				
Intercept	0.21**	0.21***	0.21***	0.22*
Time	0.004	0.004*	0.01	0.01
Weekend	0.05	0.08*	0.02	0.03
Pain	0.02	0.03	0.02	0.02
Previous day happiness	0.08*	0.07**	0.08**	0.08**
Sleep	0.000	0.001	0.000	0.0003

* *p* < .05

** *p* < .01

*** *p* < .001.

*Note*: Results from models with robust standard errors reported. Bold indicates a significant within‐person or between‐person association between negative mood and sleep. PC = person‐centered. Weekend coded 0 = weekday, 1 = weekend; Pain coded 0 = no, 1 = yes; Sex coded 0 = female, 1 = male; Race/ethnicity coded 0 = White/European American, 1 = racial/ethnic minority.

### Post hoc, exploratory analyses

Post hoc, exploratory analyses tested the extent to which associations between mood and sleep differed based on adolescents' sex, race/ethnicity, or their family's income‐to‐needs ratio (Tables S1–S4). Two significant moderation effects emerged. First, the within‐person association between daily negative mood and subjective sleep/wake problems was significantly moderated by race and ethnicity, b = 0.65, *SE* = 0.32, *p* = .041. Simple slopes analyses, using Preacher's online interaction tool for multilevel modeling (Preacher et al., 2006), indicated that the within‐person association between daily negative mood and subjective sleep/wake problems that night was stronger among adolescents of a racial/ethnic minority group, b = 1.70, *SE* = 0.23, *p* < .001, compared to White/European American adolescents, b = 0.95, *SE* = 0.25, *p* < .001. Second, the within‐person association between subjective sleep/wake problems and negative mood on the next day was significantly moderated by family income‐to‐needs ratio, b = −0.01, *SE* = 0.003, *p* = .006. Simple slopes analysis, plotted at ±1 *SD* from the mean, indicated that higher than usual subjective sleep/wake problems was associated with higher negative mood the next day for adolescents at lower levels of income‐to‐needs (i.e., lower socioeconomic status), b = 0.02, *SE* = 0.01, *p* = .03, but not for adolescents at higher levels of income‐to‐needs, b = −0.01, *SE* = 0.01, *p* = .09. Given the number of significant interactions compared to the number of interaction tests conducted across models (i.e., 3 interactions per model, 16 models), and the exploratory nature of these analyses, caution should be used in interpreting these findings.

## DISCUSSION

The goal of this study was to evaluate possible bidirectional associations between daily mood and nightly sleep in a sample of adolescents. Findings indicated support for both directions of association: Daily negative mood was associated with subjective sleep/wake problems, objective sleep efficiency, and objective sleep minutes while daily happiness was associated with subjective sleep/wake problems and objective sleep efficiency. At the same time, nightly self‐reported sleep minutes were associated with next day negative mood. The associations involving subjective sleep/wake problems were of the expected sign: negative mood was related to greater sleep/wake problems and positive mood was related to lower sleep/wake problems. However, associations involving objective measures of sleep were counter to hypotheses: negative mood was associated with higher sleep efficiency and greater objective sleep minutes and positive mood was associated with lower sleep efficiency. There were also significant, bidirectional between‐person associations (associations between variables averaged across the week‐long assessment): average negative mood was related to greater average sleep/wake problems, and average levels of happiness were related to lower average sleep/wake problems.

Associations involving self‐reported sleep/wake problems were as expected. Better mood (higher happiness and lower negative mood) predicted better quality sleep and worse mood predicted worse quality sleep. Such findings are consistent with research demonstrating that sleep and vigilance are antithetical (Nicassio et al., 1985). In order to fall asleep, persons must feel safe and enter a state of relaxation. In order to maintain vigilance to threats in the environment and consciously process information, persons must be awake. It is possible that adolescents who are experiencing negative mood or low positive mood during the day are ruminating at night, which is a state of cognitive pre‐sleep arousal in which adolescents are maintaining a state of conscious processing of thoughts. The association between depressed mood and rumination is well‐established (Hosseinichimeh et al., 2018), and rumination has also been linked to pre‐sleep arousal in young adults (Yeh et al., 2015). Future research is needed to explore emotion regulation strategies such as rumination as well as pre‐sleep arousal as explanatory mechanisms in the association between daily mood and nightly sleep.

The subjective report of sleep duration was associated with next day lower negative mood. This is also consistent with hypotheses and with prior research. For example, sleep restriction of 4 h in healthy adolescents results in significantly greater negative mood and significantly lower positive mood the next day (Reddy et al., 2017). Studies involving experimental sleep deprivation, sleep restriction, or sleep extension demonstrate neurological effects that have implications for emotional reactivity and regulation. Specifically, lower sleep duration is related to increased activity in the amygdala and reduced connectivity between the amygdala and prefrontal cortex (Yoo et al., 2007; see also Motomura et al., 2017). Thus, reduced sleep duration may enhance emotional reactivity and reduce emotion regulation abilities. In support of this, poor sleep quality predicted decreased emotion regulation (a composite of multiple strategies) in adults over a 6‐month period (O'Leary et al., 2017). In another innovative study, a full night of sleep deprivation resulted in greater negative reactions to emotional images, and reduced effectiveness of the emotion regulation strategies of cognitive reappraisal and distraction in young adults (Zhang et al., 2019).

Associations involving objective measures of adolescent sleep were not consistent with hypotheses. Negative mood during the day was related to greater objective sleep efficiency and greater sleep minutes that night. Daily happiness was related to lower objective sleep efficiency that night. There were no associations for the opposite direction of effects (i.e., sleep predicting mood the next day) that included objective measures of sleep. These findings suggest that although negative mood during the day is associated with subjectively worse sleep (greater sleep/wake problems), it is associated with objectively better sleep (longer and less disrupted sleep) that night. These findings are somewhat inconsistent with findings from previous studies of children and adolescents using objective measures of adolescent sleep. For example, Kouros and El‐Sheikh (2015) found no associations between daily mood and nightly objective sleep minutes or sleep efficiency among children who were approximately 10 years old, but more positive daily mood was associated with shorter objective sleep latency and lower physical activity during sleep. In studies of adolescents and utilizing objective measures of sleep, support for associations between sleep and mood are weak. For example, in one study, no associations between daily positive or negative mood and that night's sleep duration or efficiency were found (Doane & Thurston, 2014). In another study, Tavernier et al. (2016) only tested one direction of effects and found significant associations between negative self‐evaluative emotions and high‐arousal positive affect and worse sleep quality (longer wake bouts and longer time to fall asleep, respectively). Taken together, our findings, as well as those of previous studies to date, highlight the importance of obtaining both subjective and objective measures of sleep in order to fully understand associations between sleep and mood, especially given the study of mood and sleep among adolescents is still growing. The extent to which subjective versus objective indicators of sleep differentially predict more downstream affective and health outcomes also remains an important inquiry for future investigations.

Since the findings are novel and in an unexpected direction, it is important for them to be replicated in future studies. However, it is also important to note that previous studies on sleep and mood with adolescents, and utilizing objective measures of sleep, have had small sample sizes which may have reduced the power to detect bidirectional associations (e.g., *N* = 78, Doane & Thurston, 2015; *N* = 77, Tavernier et al., 2016), whereas the current study included 311 adolescents. It is also worth noting that there are often differential associations with subjective and objective measures of sleep. For example, McCrae et al. (2008) found associations between daily positive and negative mood and nightly sleep for subjective measures of sleep but not for objective measures of sleep in older adults. Similarly, Li et al. (2015) found a greater number of associations between daily mood and subjective rather than objective measures of women's sleep. They note that such distinct patterns of associations are common; one potential explanation is that individuals may rely on their current mood when recalling their sleep quality thereby resulting in associations between both self‐reported measures. Despite the potential for mood‐related bias in self‐reported sleep ratings, Li and colleagues argue that subjective and objective measures of sleep should be considered distinct constructs, each providing unique and important information about an individual's sleep experience.

Regarding our counter‐intuitive findings, there are several potential reasons why positive mood during the day may lead to poorer objective measures of sleep that night and negative mood during the day may lead to better objective measures of sleep that night. First, positive mood can be cognitively and physiologically arousing (El‐Sheikh & Buckhalt, 2005). A person who is very excited about an event occurring the next day, for example, may have difficulty sleeping that night. Given that adolescents experience heightened emotional reactivity, especially regarding peer interactions and social evaluations (Casey & Caudle, 2013), adolescents may be especially susceptible to such effects. Indeed, Tavernier et al. (2016) found that high arousal positive affect (excitement, energetic) during the day was associated with objectively measured longer sleep latency. Second, associations between higher than usual negative mood and higher levels of objective sleep efficiency that night may provide support for sleep as a compensatory mechanism after a particularly negative or stressful day. For example, among adults, it is posited that REM sleep physiology may attenuate amygdala and subjective emotional reactivity in response to previously encountered emotional stimuli (van der Helm et al., 2011; Walker & van der Helm, 2009). Similarly, animal studies show that social stress has been linked to subsequent greater slow wave activity during sleep (Meerlo et al., 2001). Thus, although adolescents may subjectively feel like they had poorer quality sleep, their sleep physiology may have been calibrated as a type of coping mechanism. However, actigraphy does not provide measures of sleep states and additional research is needed to explore this possibility. Third, there is some indication that persons suffering from mood disturbances have an increased likelihood of longer sleep duration. For example, moderate to severe symptoms of depression increase the odds of long sleep duration in university students across 26 countries (Peltzer & Pengpid, 2016), and long sleep duration on weekdays and weekends is associated with greater depressive symptoms in Chinese adolescents (Liu et al., 2020). Fatigue or loss of energy is a symptom of major depressive disorder based on the Diagnostic and Statistical Manual of Mental Disorders‐V (American Psychiatric Association, 2013), which may account for why negative mood is associated with more objective time spent asleep but greater subjective reports of sleep/wake problems. That is, adolescents experiencing higher than usual negative mood may feel fatigued and sleepy during day, inferring that they have not slept well. Obviously, these potential explanations are tentative and should be viewed as such pending empirical assessments.

Our study also identified several significant differences in sleep and mood between weekdays and weekends that are worth noting. Specifically, daily happiness, sleep duration, and sleep efficiency were significantly higher on weekends compared to weekdays, while negative mood and sleep/wake problems were significantly lower on weekends compared to weekdays. As weekdays are school days, it is likely that school start times are curtailing adolescent sleep periods and resulting in shorter sleep duration (Nahmod et al., 2019). Furthermore, peer interactions, teacher interactions, and academic work may be more stressful, frustrating, and distressing than the leisure activities and preferred social interactions that are more likely to occur on weekends (Weinstein & Mermelstein, 2007).

Although not the primary focus of this study, given the large and diverse sample, we were also able to test for individual differences in sleep‐daily mood associations based on demographic characteristics, including sex, race and ethnicity, and family socioeconomic status in post hoc, supplemental analyses. Two significant interactions emerged suggesting that the within‐person association between negative mood and subjective sleep/wake problems that night was stronger among adolescents who were Black/African American, Hispanic/Latinx American, or multiracial as compared to White/European American adolescents. The opposite direction of effects (i.e., within‐person association between sleep/wake problems and negative affect the next day) was significant only among adolescents from families of lower SES. Thus, although preliminary, the small number of significant findings suggest that belonging to a historically minoritized group or a family with fewer resources may exacerbate the association between sleep quality and negative affect. Children who are economically disadvantaged or historically minoritized are more likely to experience an aggregation of risk due to exposure to past, current, and continuing stressors (e.g., Fuligni et al., 2021; Yip et al., 2020). For example, due to systemic racism and structural barriers which fail to support children from economically disadvantaged homes (Schiele, 2014), they may be more likely to experience discrimination (Yip et al., 2020), live in impoverished neighborhoods (e.g., Mayne et al., 2021) and experience disruptive sleep environments (e.g., noise exposure; McWood et al., 2021). These factors may disrupt sleep quality and exacerbate the effects of poor‐quality sleep on the next day's mood. Nevertheless, with the exception of one significant interaction for each of race and SES, all of the associations between sleep and daily mood did not differ based on demographic variables attesting to the robust nature of the findings across participants.

With regard to adolescent sex, there were no significant moderation effects in any of the models. There are relatively few studies that have tested for sex differences in associations between daily mood and sleep with which to compare our findings. For example, van Zundert et al. (2015) reported that the association between self‐reported sleep quality and negative affect the next day was stronger for girls, but there were no sex differences when testing mood as a predictor of sleep the same night. Further research with large, diverse samples is needed that explicitly examine individual differences in within‐person associations between sleep and daily mood, including both risk and resilience processes.

The findings should be interpreted in light of study limitations. Associations between daily mood and nightly sleep were assessed at a single age, and it is important for future research to examine the long‐term implications of mood and sleep dynamics for mental and sleep health. Furthermore, the only measure of positive mood was one‐item assessing happiness, and a global assessment of negative mood was examined. Future research, including studies of different forms of positively (excitement vs contentment) and negatively (anxiety vs depression) arousing moods are needed. The examination of depressed mood is especially needed, as it may account for the associations observed with apparent objectively improved sleep. There are also a number of different ways in which associations between sleep and mood measured daily can be assessed. For example, future research can examine varying lagged effects of sleep disruptions, such as accumulations of sleep debt, on mood. In addition, emotional lability, or the degree to which mood fluctuates throughout the day, is an important aspect of emotional health that future research should investigate, as well as considering day‐to‐day variability in sleep duration and quality. Another important future direction is to assess individual differences in within‐person associations between sleep and mood, such as concurrent levels of stress or mental health difficulties. It is possible that associations between sleep and mood may be exacerbated among individuals whose regulatory capacities are already taxed. Finally, it is possible that associations between subjective sleep and mood are somewhat inflated due to shared method variance (both were assessed via adolescent self‐report) or mood‐congruent bias (e.g., assuming one slept well because they are in a good mood; Konjarski et al., 2018).

Despite study limitations and the need for future research, the current study sheds new light on associations between daily mood and sleep in adolescence. Our study makes a novel contribution to the literature on sleep and mood by identifying significant associations between subjective and objective measures of sleep and daily mood (although counter to hypotheses for objective measures) in a large, diverse sample of adolescents. Findings suggest that negative mood during the day is associated with subjectively worse sleep that night, but objectively longer and more efficient sleep. Similarly, daily happiness is related to subjectively better sleep that night but objectively less efficient sleep. In the reverse direction, longer subjective sleep duration was related to less negative mood the next day. While the associations with subjective sleep were as expected, results with objective sleep indicate positive mood may in some ways disrupt adolescent sleep while negative mood may lead some adolescents to spend more time sleeping. Overall, the findings provide stronger support for affect predicting sleep compared to the opposite direction of effects. In terms of clinical intervention, these findings support recent efforts to modify cognitive behavior therapy for insomnia (CBT‐I) for use with adolescents. Although numerous clinical trials show the efficacy of CBT‐I for adults, there is still only a small number of clinical trials with children and adolescents (Dewald‐Kaufmann et al., 2019). What makes CBT‐I particularly interesting in light of the current findings is that treatment components involve stimulus control, challenging negative thoughts, and relaxation training. These components, thus, provide skills that may help regulate daily affective experiences and prevent them from disrupting nightly sleep. Given the importance of the adolescent developmental period with regard to changes in both sleep and mood regulation, additional prospective studies of bidirectional associations between subjective and objective sleep and adolescent daily mood, in large diverse samples, are warranted.

## CONFLICT OF INTEREST

None.

## Supporting information


Table S1
