# Supplementary material for: Bidirectional associations between nightly sleep and daily happiness and negative mood in adolescents
Source: Child Dev. 2022 May 21;93(5):e547–62. doi: 10.1111/cdev.13798 (PMC9545079; doi:10.1111/cdev.13798)
Supplement: Supplementary file 1 — Table S1 [file CDEV-93-e547-s001.docx]

**Supplemental Table 1**

*Race/Ethnicity, Sex, and Family Income-to-Needs Ratio as Moderators of Associations between Daily Negative Mood and Sleep the Same Night*

|  | Dependent Variable: | | | |
| --- | --- | --- | --- | --- |
|  | Self-reported  Sleep Minutes | Self-reported  Sleep/Wake Problems | Objective  Sleep Minutes | Objective  Sleep Efficiency |
|  | Fixed Effects | | | |
|  | b (SE), *p* | b (SE), *p* | b (SE), *p* | b (SE), *p* |
| Intercept | 487.19 (6.50), *p* <.001 | 12.46 (0.17), *p* <.001 | 402.38 (4.85), *p*<.001 | 93.13 (0.44), *p* <.001 |
| Time | -4.14 (1.62), *p* = .011 | -0.09 (0.03), *p* = .003 | -1.15 (0.95), *p* = .23 | -0.01 (0.07), *p* = .88 |
| Weekend vs. Weekday | 42.35 (7.42), *p* <.001 | -0.56 (0.15), *p* <.001 | 27.66 (4.69), *p* <.001 | -0.35 (0.33), *p* = .30 |
| Daily Pain | -0.62 (8.73), *p* = .94 | 0.54 (0.21), *p* = .012 | -1.50 (6.51), *p* = .82 | -1.70 (0.54), *p*= .002 |
| Negative Mood_PC_ (within-person) | 0.43 (5.02), *p* = .93 | **1.33 (0.16), *p* <.001** | 6.62 (3.74), *p* = .08 | **0.71 (0.30), *p* = .018** |
| Averaged Negative Mood (between-person) | -7.89 (6.58), *p* = .23 | **2.17 (0.28), *p* <.001** | -3.06 (5.26), *p* = .56 | 0.05 (0.64), *p* = .94 |
| Sex | -14.02 (8.61), *p* = .11 | -0.15 (0.29), *p* = .60 | -23.90 (6.65), *p* <.001 | -1.68 (0.77), *p* = .031 |
| Race/Ethnicity | -24.27 (8.84), *p* = .006 | -0.34 (0.32), *p* = .29 | -32.06 (7.66), *p* <.001 | -2.45 (0.89), *p* = .006 |
| Income-to-Needs Ratio | -3.68 (2.33), *p* = .12 | 0.07 (0.10), *p* <.001 | 2.69 (1.78), *p* =.13 | 0.61 (0.20), *p* = .002 |
| Negative Mood X Sex | -0.61 (9.71), *p* = .95 | 0.28 (0.32), *p* = .38 | -5.53 (7.12), *p* = .44 | -0.03 (0.57), *p* = .96 |
| Negative Mood X Race/Ethnicity | 6.45 (9.79), *p* = .51 | **0.65 (0.32), *p* = .041** | -1.58 (6.97), *p* = .82 | 0.02 (0.64), *p* = .98 |
| Negative Mood x Income-to-Needs Ratio | 3.52 (2.83), *p* = .22 | -0.06 (0.09), *p* = .47 | -0.91 (1.81), *p* = .62 | 0.10 (0.18), *p* = .58 |

*Note*. Results from models with robust standard errors reported. Bold indicates a significant within-person or between-person association between daily mood and sleep, or a significant cross-level interaction. Weekend coded 0 = weekday, 1 = weekend; Pain coded 0 = no, 1 = yes; Sex coded 0 = female, 1 = male; Race/ethnicity coded 0 = White, 1 = racial/ethnic minority. **p*<.05, ***p*<.01, ****p*<.001

**Supplemental Table 2**

*Race/Ethnicity, Sex, and Family Income-to-Needs Ratio as Moderators of Associations between Daily Happiness and Sleep the Same Night*

|  | Dependent Variable: | | | |
| --- | --- | --- | --- | --- |
|  | Self-reported  Sleep Minutes | Self-reported  Sleep/Wake Problems | Objective  Sleep Minutes | Objective  Sleep Efficiency |
|  | Fixed Effects | | | |
|  | b (SE), *p* | b (SE), *p* | b (SE), *p* | b (SE), *p* |
| Intercept | 487.46 (6.54), *p* <.001 | 12.42 (0.18), *p* <.001 | 401.94 (4.86), *p* <.001 | 93.12 (0.44), *p* |
| Time | -4.07 (1.64), *p* = .014 | -0.09 (0.03), *p* = .003 | -1.08 (0.95), *p* = .25 | -0.01 (0.07), *p* = .91 |
| Weekend vs. Weekday | 42.00 (7.47), *p* <.001 | -0.56 (0.15), *p* <.001 | 28.31 (4.70), *p* <.001 | -0.43 (0.33), *p* = .19 |
| Daily Pain | -0.53 (8.54), *p* = .95 | 0.88 (0.23), *p* <.001 | -0.28 (6.52), *p* = .97 | -1.53 (0.54), *p* = .005 |
| Daily Happiness_PC_ (within-person) | 3.44 (3.77), *p* = .36 | **-0.91 (0.10), *p* <.001** | **-**5.09 (2.84), *p* = .07 | -0.47 (0.24), = .048 |
| Averaged Happiness (between-person) | 1.23 (7.73), *p* = .87 | **-1.71 (0.25), *p* <.001** | -2.45 (5.44), *p* = .65 | 0.05 (0.53), *p* = .93 |
| Sex | -12.64 (8.39), *p* = .13 | -0.48 (0.29), *p* = .10 | -22.74 (6.57), *p* <.001 | -1.68 (0.76), *p* = .027 |
| Race/Ethnicity | -22.30 (8.78), *p* = .012 | -0.81 (0.33), *p* = .014 | -30.63 (7.46), *p* <.001 | -2.39 (0.86), *p* = .006 |
| Income-to-Needs Ratio | -3.34 (2.36), *p* = .16 | 0.05 (0.10), *p* = .62 | 2.99 (1.82), *p* = .10 | 0.61 (0.19), *p* = .001 |
| Positive Mood X Sex | 2.58 (7.30), *p* = .72 | -0.37 (0.20), *p* = .07 | -4.19 (5.40), *p* = .44 | -0.40 (0.48), *p* = .40 |
| Positive Mood X Race/Ethnicity | -7.15 (7.50), *p* = .34 | -0.09 (0.21), *p* = .69 | 0.07 (5.87), *p* = .99 | -0.38 (0.48), *p* = .43 |
| Positive Mood x Income-to-Needs Ratio | 1.13 (2.25), *p* = .62 | -0.10 (0.06), *p* = .09 | -1.31 (1.71), *p* = .44 | 0.10 (0.13), *p* = .42 |

*Note*. Results from models with robust standard errors reported. Bold indicates a significant within-person or between-person association between daily mood and sleep, or a significant cross-level interaction. Weekend coded 0 = weekday, 1 = weekend; Pain coded 0 = no, 1 = yes; Sex coded 0 = female, 1 = male; Race/ethnicity coded 0 = White, 1 = racial/ethnic minority. **p*<.05, ***p*<.01, ****p*<.001

**Supplemental Table 3**

*Race/Ethnicity, Sex, and Family Income-to-Needs Ratio as Moderators of Associations between Sleep and Negative Mood the Next Day*

|  | Dependent Variable: Negative Mood | | | | |
| --- | --- | --- | --- | --- | --- |
|  | Self-reported  Sleep Minutes  Predicting Negative Mood | Self-reported  Sleep/Wake Problems Predicting Negative Mood | Objective  Sleep Minutes  Predicting Negative Mood | Objective  Sleep Efficiency  Predicting Negative Mood |  |
|  | Fixed Effects | | | | |
|  | b (SE), *p* | b (SE), *p* | b (SE), *p* | b (SE), *p* |  |
| Intercept | 1.90 (0.04), *p* <.001 | 1.88 (0.03), *p* <.001 | 1.90 (0.09), *p* <.001 | 1.91 (0.03), *p* <.001 |  |
| Time | -0.05 (0.01), *p* <.001 | -0.05 (0.01), *p* <.001 | -0.05 (0.01), *p* <.001 | -0.05 (0.01), *p* <.001 |  |
| Weekend vs. Weekday | -0.07 (0.03), *p* = .027 | -0.08 (0.03), *p* = .006 | -0.09 (0.03), *p* = .008 | -0.09 (0.03), *p* = .004 |  |
| Daily Pain | 0.09 (0.05), *p* = .07 | 0.09 (0.05), *p* = .08 | 0.08 (0.05), *p* = .16 | 0.08 (0.05), *p* = .014 |  |
| Previous Day Negative Mood | 0.20 (0.03), *p* <.001 | 0.15 (0.03), *p* <.001 | 0.24 (0.03), *p* <.001 | 0.24 (0.03), *p* <.001 |  |
| Sleep_PC_ (within-person) | **-0.003 (0.0002), *p* = .039** | 0.002 (0.005), *p* = .70 | -0.0003 (0.0002), *p* = .24 | -0.001 (0.003), *p* = .77 |  |
| Averaged Sleep (between-person) | -0.0002 (0.0003), *p* = .59 | **0.07 (0.01), *p* <.001** | -0.0001 (0.0004), *p* = .90 | 0.002 (0.004), *p* = .55 |  |
| Sex | -0.20 (0.05), *p* <.001 | -0.18 (0.05), *p* <.001 | -0.21 (0.05), *p* <.001 | -0.22 (0.05), *p* <.001 |  |
| Race/Ethnicity | -0.21 (0.06), *p* <.001 | -0.13 (0.05), *p* = .014 | -0.19 (0.06), *p*= .002 | -0.19 (0.06), *p* = .002 |  |
| Income-to-needs Ratio | -0.03 (0.01), *p* = .022 | -0.02 (0.01), *p* = .06 | -0.03 (0.01), *p* = .014 | -0.04 (0.01), *p* = .012 |  |
| Sleep X Sex | 0.0004 (0.0003), *p* = .19 | 0.003 (0.01), *p* = .76 | 0.001 (0.0004), *p* = .18 | 0.0003 (0.01), *p* = .95 |  |
| Sleep x Race/Ethnicity | -0.0001 (0.0004), *p* = .73 | -0.01 (0.01), *p* = .40 | 0.001 (0.001), *p* = .09 | -0.002 (0.01), *p* = .77 |  |
| Sleep x Income-to-Needs Ratio | -0.0001 (0.0001), *p* = .13 | **-0.01 (0.003), *p* = .006** | 0.00004 (0.0001), *p* = .76 | -0.0001 (0.002), *p* = .98 |  |

*Note*. Results from models with robust standard errors reported. Bold indicates a significant within-person or between-person association between daily mood and sleep, or a significant cross-level interaction. Weekend coded 0 = weekday, 1 = weekend; Pain coded 0 = no, 1 = yes; Sex coded 0 = female, 1 = male; Race/ethnicity coded 0 = White, 1 = racial/ethnic minority. **p*<.05, ***p*<.01, ****p*<.001

**Supplemental Table 4**

*Race/Ethnicity, Sex, and Family Income-to-Needs Ratio as Moderators of Associations between Sleep and Daily Happiness the Next Day*

|  | Dependent Variable: Daily Happiness | | | | |
| --- | --- | --- | --- | --- | --- |
|  | Self-reported  Sleep Minutes  Predicting Happiness | Self-reported  Sleep/Wake Problems Predicting Happiness | Objective  Sleep Minutes  Predicting Happiness | Objective  Sleep Efficiency  Predicting Happiness |  |
|  | Fixed Effects | | | | |
|  | b (SE), *p* | b (SE), *p* | b (SE), *p* | b (SE), *p* |  |
| Intercept | 3.71 (004), *p* <.001 | 3.72 (0.04), *p* <.001 | 3.52 (0.12), *p* <.001 | 3.71 (0.05), *p* <.001 |  |
| Time | 0.003 (0.01), *p* = .81 | 0.005 (0.01), *p* = .63 | -0.002 (0.01), *p* = .89 | -0.002 (0.01), *p* = .87 |  |
| Weekend vs. Weekday | 0.10 (0.04), *p* = .027 | 0.10 (0.04), *p* = .019 | 0.11 (0.04), *p* = .01 | 0.11 (0.04), *p* = .01 |  |
| Daily Pain | -0.13 (0.06), *p* = .032 | -0.10 (0.06), *p* = .10 | -0.12 (0.06), *p* = .06 | -0.13 (0.06), *p* = .051 |  |
| Previous Day Happiness | 0.11 (0.03), *p* <.001 | 0.06 (0.03), *p* = .054 | 0.13 (0.03), *p* <.001 | 0.12 (0.03), *p* <.001 |  |
| Sleep_PC_ (within-person) | 0.00005 (0.0002), *p* = .83 | 0.004 (0.01), *p* = .60 | 0.0001 (0.0003), *p* = .79 | 0.001 (0.004), *p* .78 |  |
| Averaged Sleep (between-person) | -0.0001 (0.0005), *p* = .80 | **-0.07 (0.01), *p* <.001** | -0.001 (0.001), *p* = .09 | -0.01 (0.01), *p* = .23 |  |
| Sex | 0.08 (0.07), *p* = .24 | 0.06 (0.06), *p* = .39 | 0.06 (0.07), *p* = .40 | 0.08 (0.07), *p* = .26 |  |
| Race/Ethnicity | 0.06 (0.07), *p* = .43 | 0.0005 (0.07), *p* = .99 | 0.02 (0.07), *p* = .82 | 0.04 (0.08), *p* = 64 |  |
| Income-to-needs Ratio | 0.05 (0.02), *p*= .002 | 0.05 (0.02), *p* = .012 | 0.05 (0.023), *p* = .002 | 0.05 (0.02), *p* = .002 |  |
| Sleep X Sex | -0.0003 (0.0004), *p* = .44 | 0.002 (0.01), *p* = .91 | -0.001 (0.001), *p* = .24 | -0.003 (0.01), *p* = .71 |  |
| Sleep x Race/Ethnicity | 0.0008 (0.0005), *p* = .08 | -0.001 (0.02), *p* = .97 | 0.001 (0.001), *p*= .34 | -0.004 (0.01), *p* = .64 |  |
| Sleep x Income-to-Needs Ratio | 0.0002 (0.0001), *p* = .08 | 0.004 (0.003), *p* = .20 | 0.0002 (0.0001), *p* = .12 | 0.001 (0.002), *p* = .56 |  |

*Note*. Results from models with robust standard errors reported. Bold indicates a significant within-person or between-person association between daily mood and sleep, or a significant cross-level interaction. Weekend coded 0 = weekday, 1 = weekend; Pain coded 0 = no, 1 = yes; Sex coded 0 = female, 1 = male; Race/ethnicity coded 0 = White, 1 = racial/ethnic minority. **p*<.05, ***p*<.01, ****p*<.001
